# Arrhythmias and Sudden Cardiac Death in Beta-Thalassemia Major Patients: Noninvasive Diagnostic Tools and Early Markers

**DOI:** 10.1155/2019/9319832

**Published:** 2019-11-30

**Authors:** Vincenzo Russo, Enrico Melillo, Andrea A. Papa, Anna Rago, Celeste Chamberland, Gerardo Nigro

**Affiliations:** ^1^Chair of Cardiology, Department of Translational Medical Sciences, University of Campania “Luigi Vanvitelli”, Monaldi Hospital, Naples, Italy; ^2^Department of History and Philosophy, Roosevelt University, Chicago, Illinois, USA

## Abstract

Beta-thalassemias are a group of inherited, autosomal recessive diseases, characterized by reduced or absent synthesis of beta-globin chains of the hemoglobin tetramer, resulting in variable phenotypes, ranging from clinically asymptomatic individuals to severe anemia. Three main forms have been described: heterozygotes, homozygotes *β*+, and homozygotes *β*°. Beta-thalassemia major (*β*-TM), the most serious form, is characterized by an absent synthesis of globin chains that are essential for hemoglobin formation, causing chronic hemolytic anemia. Cardiac complications represent a leading cause of mortality in *β*-TM patients, although an important and progressive increase of life expectancy has been demonstrated after the introduction of chelating therapies. Iron overload is the primary factor of cardiac damage resulting in thalassemic cardiomyopathy, in which diastolic dysfunction usually happens before systolic impairment and overt heart failure (HF). Although iron-induced cardiomyopathy is slowly progressive and it usually takes several decades for clinical and laboratory features of cardiac dysfunction to manifest, arrhythmias or sudden death may be present without signs of cardiac disease and only if myocardial siderosis is present. Careful analysis of electrocardiograms and other diagnostic tools may help in early identification of high-risk *β*-TM patients for arrhythmias and sudden cardiac death.

## 1. Introduction

Beta-thalassemia major (*β*-TM) is a genetic hemoglobin disorder characterized by a reduced or absent synthesis of globin chains that are essential for hemoglobin formation, causing chronic hemolytic anemia. Three main forms have been described: heterozygotes, homozigotes *β*+, and homozygotes *β*° [1]. Clinical management of thalassemia major consists of regular long-life red blood cell transfusions and iron chelation therapy to remove iron introduced in excess with transfusions. Iron deposition in combination with inflammatory and immunogenic factors is involved in the pathophysiology of cardiac dysfunction in these patients. Myocardial iron overload leads to heart failure (HF) and arrhythmias, which are the most important life-threatening complications of *β*-TM patients. Arrhythmias or sudden cardiac death may be present in an early stage of iron overload cardiomyopathy, without overt signs and symptoms of cardiac disease [2]. The aim of this review is to describe the most common arrhythmic disorders in *β*-TM patients and to provide an overview about risk markers and noninvasive diagnostic tools to identify *β*-TM patients at a higher risk of arrhythmias and sudden cardiac death.

## 2. Pathophysiology of Iron Cardiac Toxicity and Cardiac Arrhythmias

The primary factor of cardiac damage in *β*-TM is iron overload [3–5], which has both direct and indirect toxic effects. Iron overload results primarily from repetitive blood transfusions, as well as by hemolysis and increased intestinal absorption. In *β*-TM patients, iron deposition in parenchymal tissues begins within 1 year of starting regular transfusions. When the iron transfer capacities of transferrin are exceeded, as in the case of iron overload, a nontransferrin-bound form of iron appears in the blood. Nontransferrin-bound free iron, which is capable of generating toxic oxygen free radicals, is immediately buffered by the cytosolic ferritin, degraded to hemosiderin, and stored in the lysosomes. When the intracytosolic buffering mechanisms fail, toxic labile iron levels rise in the cardiomyocyte, resulting in oxidative damage to cell membranes and ion channels. The free iron catalyzes the formation of hydroxyl radical, is highly reactive, and attacks lipids, proteins and DNA, which ultimately leads to cell death and fibrosis [4–6]. Oxidative stress-mediated iron toxicity also increases the lysosomal fragility [7] and decreases mitochondrial inner membrane respiratory enzyme activity, [8] protective antioxidant enzyme activity, myofibril elements, and number of mitochondria [9]. Iron overloaded cardiomyocytes have a smaller overshoot potential and shorter action potential duration than iron-free cardiomyocytes in the same heart [10]. An alteration in ion currents characterized by reduced Na+ currents may be an underlying mechanism [11]. Reduced overshoot potential ensues as a result of decreased rapid phase 0 depolarization (fast sodium current). A reduction in the late fast sodium current during the plateau phase may result in the rapid shortening of the action potential duration because of the disturbance of a delicate balance of small currents. Another postulated mechanism for the electrophysiologic effect of iron overload is the blockage of ryanodine calcium channels and oxidative stress-mediated changes in sarcoplasmic calcium release and reuptake [12]. This electrophysiological heterogeneity, including the patchy nature of cardiac iron deposition, may provide the substrate for triggered and re-entry activity, and it may be involved in the genesis of arrhythmias and cardiac complications in *β*-TM patients [13–15].

## 3. Cardiac Arrhythmias and Sudden Cardiac Death

Supraventricular ectopic beats or no-sustained tachycardia may be present in the earlier stage of the *β*-TM cardiomyopathy, while malignant arrhythmias are usually present in the advanced stage of the disease. The mismatch between symptoms and the severity of arrhythmias in *β*-TM patients may lead to a delayed diagnosis of life-threatening arrhythmias and might explain the high incidence of sudden cardiac death reported in the literature [16].

Atrial fibrillation (AF), atrial flutter (AFl), and intra-atrial re-entrant tachycardia are the most common clinically relevant rhythm disturbances in *β*-TM patients. Ectopic atrial tachycardia and chaotic atrial rhythm may also be seen, particularly in the presence of significant iron loading [17, 18]. Ventricular arrhythmias are more specific for iron cardiotoxicity, and the presence of couplets and nonsustained ventricular tachycardia should raise clinical suspicion [19].

The precise incidence of cardiac arrhythmias in the *β*-TM population is still challenged. A study including 481 *β*-TM patients, enrolled in the Myocardial Iron Overload in Thalassemia (MIOT) project from 57 Italian thalassemia centers, showed a lower incidence of cardiac arrhythmias (3.2%) compared with that (14%) of a historical cohort of *β*-TM patients [7, 20].

This difference can primarily be explained by the increasing diagnostic and predictive value of T2∗ (T2 star) CMR (Cardiac Magnetic Resonance) in *β*-TM patients, which was not generally recognized in the early 2000s, and that probably led to a different management of chelation therapy in response to iron overload assessment. Moreover, these studies employed different chelation regimens; the availability in the clinical practice of new iron chelators has allowed physicians to apply progressively a tailored pharmacological approach [20].

To date, little is known about the true incidence of AF in *β*-TM patients, because there are many heterogeneous studies including small numbers of patients, with different clinical characteristics, investigated using various methods for AF detection [21].

AF is a common finding on 24-hour Holter monitoring in *β*-TM patients without cardiac dysfunction with a prevalence up to 20% when a prolonged electrocardiographic monitoring by external loop recorder was used for the diagnosis [22, 23]. The early detection of AF in *β*-TM patients is of pivotal importance for the management of clinical follow-up and for the optimization of medical therapy, in order to evaluate the opportunity of prophylactic anticoagulation treatment or nonpharmacologic approaches for stroke prevention [24].

*β*-TM patients are also at increased risk for malignant arrhythmias and sudden cardiac death (SCD). Historically, SCD accounts for about 5% of cardiac deaths and is associated with severe iron overload and increased QT dispersion (QTd), which suggests iron-mediated repolarization abnormalities and torsades de pointes as a causative mechanism [25–28].

Although the majority of SCD occurs in *β*-TM patients with end-stage thalassemic cardiomyopathy [29], our recent study suggested a high incidence of SCD among young patients without clinical evidence of cardiac disease, with a 27% occurrence rate over a 26-year observation period [16]. Sudden cardiac death due to bradiarrhythmias and complete heart block was relatively common in thalassemic patients before the availability of chelation therapy, occurring in up to 40% of those aged over 15 years [30]. Today, it is rarely reported in most communities, but may occasionally be encountered in the context of advanced thalassemic cardiomyopathy with severe iron overload.

## 4. Diagnostic Workup and Arrhythmic Risk Markers

The arrhythmic risk evaluation in *β*-TM patients without evidence of cardiac disease remains a major clinical challenge [2]. The 12-lead resting ECG is the most frequently used examination tool for the evaluation of patients with cardiovascular disease and, because of its relatively low cost, to date it represents the gold standard for the screening [31].

Electrocardiographic abnormalities have been well documented in both the pre- and postchelation era [32, 33]. New-onset electrocardiographic abnormalities are usually evident in *β*-TM patients with HF and may include electrocardiographic findings that suggest left-sided heart (Q1S3 pattern and extreme left-axis deviation) or right-sided heart involvement (S1Q3 pattern and right-axis deviation), T-wave inversion beyond lead V1, P-wave prolongation or abnormalities and a consistent decrease in QRS amplitude. An abnormal ECG was found in 46% of *β*-TM patients without HF (T-wave abnormalities in 34% and right bundle-branch block in 12%) [34].

**Figure 1:**
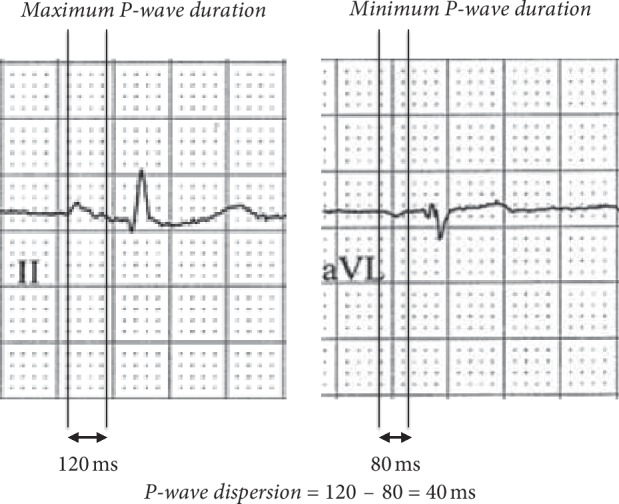
P-wave dispersion measured as difference between (a) maximum P-wave duration (P max) and (b) minimum P-wave duration (P min) at 12-lead surface ECG. In this case, the P max was observed in DII and the P min was observed in AVL.

The prognostic value of P-wave duration and P-wave dispersion (PD) in *β*-TM patients with conserved cardiac function has been previously investigated [22]. PD is defined as the difference between the maximum P-wave duration (P max) and the minimum P-wave duration on twelve leads ECG (Figure 1). In a 12-month follow-up, a cut-off value of 111 ms for P max had a sensitivity of 80% and a specificity of 87%, and a cut-off value of 35.5 ms for PD had a sensitivity of 90% and a specificity of 85% for early detection of new onset of AF episodes in *β*-TM patients [22]. The association between PD and increased risk of atrial fibrillation also has been confirmed in other clinical conditions [35, 36] . The ECG ability to predict silent AF may identify a group of beta-thalassemia patients whose thromboembolic stroke risk can be modified with anticoagulant therapy. In fact, cardioembolic stroke has been reported in 0.25–0.46% of patients with *β*-TM in different endemic countries [37]. While patients with sickle *β*-thalassemia and thalassemia intermedia present asymptomatic ischemic lesions that spare the cortex [38], *β*-TM patients seem to suffer large hemispheric territorial infarcts in the presence of atrial fibrillation (AF) and cardiomyopathy [39].

An increased dispersion of ventricular repolarization is considered to provide the electrophysiological substrate for life-threatening ventricular arrhythmias in several clinical conditions [40–42]. *β*-TM patients usually showed increased QTc intervals and increased indexes of ventricular repolarization heterogeneity such as QT dispersion (QTd), JT dispersion (JTd), and T peak-to-end/QT ratios, both strictly related to serum ferritin levels [43]. *β*-TM patients who experienced sudden cardiac death had a higher value of QTd and JTd to the baseline ECGs than the cohort who survived. In particular, a QTd cut-off value >70 ms and JTd cut-off >100 ms identified high-risk sudden death *β*-TM patients who need careful cardiac monitoring [16].

In addition, *β*-TM patients exhibited attenuated heart rate variability (HRV) at the first and second minute of recovery after exercise stress test compared with controls [43]. HRV is considered a reliable method to assess the autonomic nervous system fluctuations in normal healthy individuals or in patients with various cardiovascular and noncardiovascular disorders; in particular, a decreased HRV reflects enhanced sympathetic overdrive and decreased vagal activity and has a strong association with the pathogenesis of cardiac arrhythmias [44–46].

Increased QRS duration, even within normal limits, predicts mortality in the general population, and QRS fragmentation (fQRS) is associated with increased risk of arrhythmic events and mortality in a variety of cardiac disorders [47–50].

*β*-TM population also showed an increased QRS duration and QRS fragmentation, which correlates with the myocardial iron overload assessed with MRI T2∗, compared with healthy controls [51]. These findings support the hypothesis that myocardial iron overload causes abnormal and nonhomogeneous myocardial repolarization which may be responsible for electrocardiographic abnormalities. Therefore, it is reasonable to assume that early detection of arrhythmic risk, through a careful ECG analysis, may have significant clinical impact in the management of these patients.

Besides basic and advanced ECG analysis, the transthoracic echocardiography (TTE) represents a useful noninvasive imaging technique to stratify the arrhythmic risk in *β*-TM patients. The advanced stage of thalassemic cardiomyopathy is characterized by restrictive diastolic dysfunction that usually occurs before systolic dysfunction with an increased risk of ventricular arrhythmias [52]. However, in the earlier stages when cardiac function is preserved, TTE and Doppler techniques may provide useful parameters for the arrhythmic risk evaluation.

In a multiparametric study by Hamed et al., which assessed the standard M-mode echocardiographic measure, cardiac MRI T2∗, ECG stress test, and 24 h ECG Holter monitoring in a *β*-TM cohort, patients who developed arrhythmias had significantly higher values of left atrial diameter (LAD), interventricular septum diameter (IVSd), and LV posterior wall diameter (LVPWd). Furthermore, LAD, IVSd, and LVPWd were significantly negatively correlated with the cardiac T2∗ [53].

Left atrial mechanical function and left ventricle diastolic dysfunction seems to be strictly related to the risk of AF development and to neurohumoral response in *β*-TM patients [54]. Risk factors for new onset AF were elevated *E*/*E*′ ratio (an index of elevated left ventricle diastolic pressure) and decreased passive and active atrial function, while there was no correspondence between AF occurrence and MRI T2∗. Left atrial function, assessed through the left atrial active and passive emptying fraction (LAPEF and LAEF), was reduced in *β*-TM patients before the diastolic dysfunction or subclinical left ventricle systolic dysfunction occurred. These results strengthen the central role of left atrium mechanics in the pathogenesis of supraventricular arrhythmias. Many factors like volume overload from hyperkinetic circulation and anemia, high diastolic pressures, and atrial hemosiderosis all lead to fibrosis and participate in mechanisms of electrical remodeling and AF generation [55, 56].

Monte et al. [57] evaluated the relationship between Color Doppler Myocardial Imaging (CDMI) and the development of supraventricular arrhythmias in a 23 *β*-TM patients cohort. Nine patients developed supraventricular arrhythmias, and once again LV, LA dimensions, and *E*/*E*′ ratio were significantly higher in this group. CDMI-derived strain and strain rates, a subclinical marker of impaired myocardial deformation, were lower than in controls in all *β*-TM patients. Early and late diastolic strain rate peak at IVS and LV wall level was significantly lower in arrhythmic patients as well as strain and strain rate values at LA lateral wall. Thus, a reduced myocardial deformation assessed with LA and LV strain can provide useful tools not only for arrhythmic risk stratification but also for early detection of iron overload cardiomyopathy.

**Figure 2:**
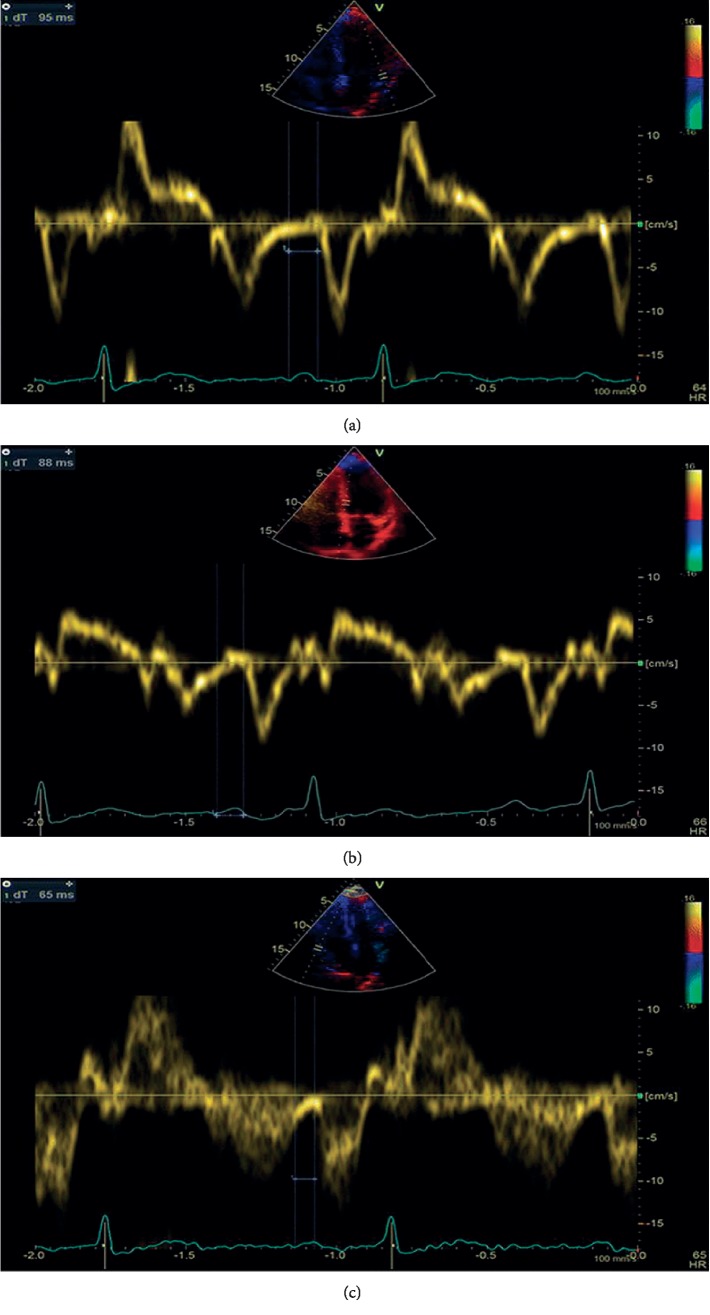
Atrial electromechanical delay: PA interval is measured from the onset of P-wave on surface ECG to the beginning of A-wave on tissue doppler imaging, with sample volume placed at the lateral mitral annulus (a); at septal mitral annulus (b); and right ventricular tricuspid annulus (c).

Our previous study evaluated the role of the atrial electromechanical delay (AEMD) in identifying *β*-TM patients at an increased risk to develop AF during a 5-year follow-up [23]. AEMD is the time interval from the onset of P-wave on surface ECG to the beginning of the late diastolic wave on Tissue Doppler Imaging (TDI) from lateral mitral annulus (L-PA), septal mitral annulus (S-PA), and right ventricular (RV) tricuspid annulus (RV-PA). The difference between septal PA and RV PA is defined as intraright AEMD, the difference between L-PA and S-PA is defined as intraleft AEMD, and the difference between L-PA and RV-PA is defined as inter-AEMD. (Figure 2). AEMD has been associated with an increased risk of atrial fibrillation in some clinical conditions [58–61]. A cut-off value of 40.1 ms for intraleft AEMD had a sensitivity of 76.2% and a specificity of 97.5% in identifying *β*-TM patients with AF risk. A cut-off value of 44.8 ms for inter-AEMD had a sensitivity of 81.2% and a specificity of 98.7% in identifying *β*-TM patients with AF risk [23].

**Figure 3:**
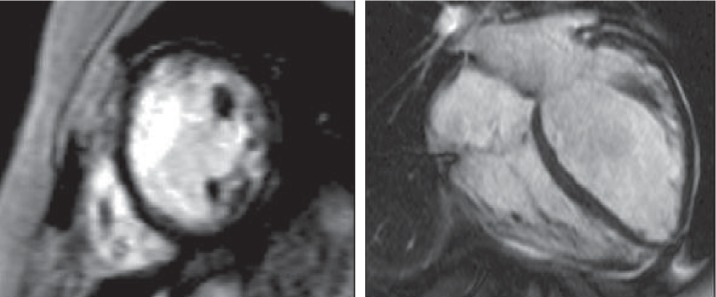
Cardiac T2∗ (T2 star) magnetic resonance showing severe myocardial iron overload.

The management of iron overload in *β*-TM has changed since the implementation in clinical practice of T2∗ CMR, which allows detection of baseline myocardial iron overload and monitoring of response during chelation therapy [62]. T2∗ is a magnetic relaxation property of any tissue and is inversely related to intracellular iron stores. Thus, T2∗ CMR gradient-echo sequences (Figure 3) and relaxation time are strongly related with the amount of myocardial iron overload with 20 ms considered as a cut-off of normality [63]. T2∗ CMR has showed a significant prognostic role in *β*-TM patients. In a prospective analysis of 652 *β*-TM patients, 98% of patients who developed HF had a T2∗ <10 ms and 83% of patients with T2∗< 20 ms developed cardiac arrhythmias [7]. The pattern and distribution of cardiac iron seems to be related with risk of HF development, since a homogeneous iron overload in all 16 cardiac segments is a predictive factor of HF [20]. However, strong evidence suggests that myocardial iron overload is less correlated with the development of arrhythmias than to heart failure [7, 64]. Furthermore, in the widespread CMR era, arrhythmias are mostly of supraventricular in origin, and left atrial dilatation, rather than myocardial iron overload, has emerged as an independent prognosticator of arrhythmias development [20].

## 5. Conclusion

Despite the advances in the management of *β*-TM, heart disease remains the leading cause of mortality in these patients. Cardiac arrhythmias are frequent in *β*-TM patients, particularly in the advanced stage of the disease, when a significant cardiac iron loading is present. The cardiovascular evaluation of *β*-TM patients should be performed by cardiologists, with experience in clinical arrhythmology and echocardiography, who have knowledge of thalassemia and iron-related cardiotoxicity. The ECG analysis should include the measuremtent of P wave and QT interval dispersion; the echocardiogram should include the evaluation of the atrial electromechanical delay or left atrial function analysis. Moreover T2∗ CMR plays a key role in the management *β*-TM patients, assessing basal myocardial iron overload and monitoring response to chelation therapy. These noninvasive tools may help to identify *β*-TM patients at high risk of atrial fibrillation onset or sudden cardiac death, even when cardiac function is conserved. For the subgroups of *β*-TM patients at high risk of developing cardiac arrhytmias, we suggest seriate 24-hours ECG Holter monitoring or cardiac loop recordings to early detect atrial fibrillation or malignant arrhythmias and to evaluate the opportunity of prophylactic treatment.
